# The Trajectory of Truth: A Longitudinal Study of the Illusory Truth Effect

**DOI:** 10.5334/joc.161

**Published:** 2021-06-08

**Authors:** Emma L. Henderson, Daniel J. Simons, Dale J. Barr

**Affiliations:** 1 Faculty of Business and Social Sciences, Kingston University, Kingston Hill Campus, Kingston Hill, Kingston upon Thames, KT2 7LB, UK; 2 School of Psychology, University of Surrey, Guildford, Surrey, GU2 7XH, UK; 3 Department of Psychology, University of Illinois at Urbana-Champaign, US; 4 Institute of Neuroscience & Psychology, University of Glasgow, UK

**Keywords:** illusory truth, repetition, truth judgement, longitudinal, Registered Report

## Abstract

Repeated statements are rated as subjectively truer than comparable new statements, even though repetition alone provides no new, probative information (the *illusory truth effect)*. Contrary to some theoretical predictions, the illusory truth effect seems to be similar in magnitude for repetitions occurring after minutes or weeks. This Registered Report describes a longitudinal investigation of the illusory truth effect (*n* = 608, *n* = 567 analysed) in which we systematically manipulated intersession interval (immediately, one day, one week, and one month) in order to test whether the illusory truth effect is immune to time. Both our hypotheses were supported: We observed an illusory truth effect at all four intervals (overall effect: *χ*^2^(1) = 169.91; *M*_repeated_ = 4.52, *M*_new_ = 4.14; H1), with the effect diminishing as delay increased (H2). False information repeated over short timescales might have a greater effect on truth judgements than repetitions over longer timescales. Researchers should consider the implications of the choice of intersession interval when designing future illusory truth effect research.

Human judgements are influenced not only by the informational value of the content we experience, but also by our subjective experience of information processing (Alter & Oppenheimer, 2009). When judging truth or accuracy, people rate repeated statements as subjectively truer than comparable new statements (the *illusory truth effect* or *repetition-induced truth effect*), even though repetition alone provides no new, probative information. That is, repetition generates the illusion of epistemic weight. Inferring truth from repetition is apposite in a world where most of the information people encounter is true, but repetition can create an illusion of truth for false information; the truth effect occurs for both true and false statements (Brown & Nix, 1996), and for both plausible and implausible ones (Fazio, Rand, & Pennycook, 2019). The effect seems robust to individual differences in cognitive ability (De keersmaecker et al., 2019). It persists even when participants are warned to avoid it (Nadarevic & Aßfalg, 2017), possess knowledge about the factual answer (Fazio, Brashier, Payne, & Marsh, 2015), or are explicitly informed about which statements are true and which are false (Begg, Anas, & Farinacci, 1992; Gilbert, Krull, & Malone, 1990; Skurnik, Yoon, Park, & Schwarz, 2005). Repeatedly reading misinformation might even reduce how unethical it feels to share that unambiguously false information on social media (Effron & Raj, 2019).

The illusory truth effect could bolster the tactics of propagandists, allowing them to amplify the believability of their message whether or not it is true (see Pennycook, Cannon, & Rand, 2018; Polage, 2012). Simply by repeating a statement, such as “no country currently has a functioning track and trace app” as Boris Johnson has said, or “President Barack Obama was born in Kenya” as Donald Trump persisted, a politician can increase belief in inaccurate or misleading information. Similarly, an advertiser can repeat scientifically spurious claims as a means to increase belief in their product’s effectiveness. Deliberate use of the illusory truth effect could amplify the believability of claims, from minor (Listerine prevents sore throats) to monumental (Iraq has weapons of mass destruction). Given the ramifications of repetition for belief, there is both a theoretical and ethical imperative to understand better the parameters of the effect.

## Explanations, Predictions and Contradictions

In the standard illusory truth effect paradigm, a set of statements, half true and half false, are presented during an exposure phase. There then follows an intersession interval that varies in length from zero minutes to several weeks. During the subsequent test phase, participants rate the perceived truth of a mix of both repeated statements and previously unseen ones. The illusory truth effect is measured by comparing truth ratings for repeated versus new statements. All explanations of the illusory truth effect, including recognition, familiarity, and the most commonly accepted explanation – processing fluency – are closely related, rely on memory, and predict that the effect should vary over time. It is perhaps surprising, then, that research to date finds little evidence that the time between repetitions (i.e., the intersession interval) changes the effect (see Dechêne, Stahl, Hansen, & Wänke, 2010). The aim of our research was to systematically manipulate intersession interval in order to test whether the illusory truth effect is unaffected by the time between repetitions. We next briefly consider the potential mechanisms underlying the illusory truth effect because, even though the Dechêne et al. meta-analysis found little evidence for an effect of delay, all of the proposed mechanisms for the illusory truth effect predict such an interaction between intersession interval and the size of the illusory truth effect. Note, though, that our study did not attempt to distinguish among the possible mechanisms. Instead, our study and the core of our literature review below focuses on the effect of interval duration.

### Recognition and familiarity

Repetition is the key prerequisite for the illusory truth effect. The perception of repetition and explicit memory for the prior presentation might enhance the effect (Bacon, 1979; Hawkins & Hoch, 1992; Law, Hawkins, & Craik, 1998). In fact, the perception that a statement has been repeated might be more important than the repetition itself (Bacon, 1979; Hawkins & Hoch, 1992; Law et al., 1998). If a statement is familiar enough for readers to think that they read it before, then they are more likely to judge it to be true. With a longer delay, people should be less likely to detect the repetition (see Brown & Nix, 1996), and the feeling of familiarity should fade (Arkes, Boehm, & Xu, 1991), hence these mechanisms predict a reduced effect with longer delays.

### Processing fluency

The predominant explanation for the illusory truth effect is fluency: Repetition leads to easier and more fluent processing. For example, semantic priming leads to faster and more accurate responses (Alter & Oppenheimer, 2009; Whittlesea, 1993). We experience fluency for stimuli we have seen recently, frequently, or for a prolonged time (Oppenheimer, 2008), and people might associate that fluency with truth (Oppenheimer, 2008; Schwarz & Jalbert, 2020). This mechanism rests on the idea that people will misattribute the fluency that comes from repetition to the informativeness or accuracy of the content, thereby increasing belief. Since fluency should be greater for recently repeated items, the illusory truth effect should decline with longer delays. If the source of fluency (in this case repetition) is conspicuous, however, people may correctly attribute their fluency to the repetition rather than to the content, eliminating the effect of repetition on truth judgments (Alter & Oppenheimer, 2009; Nadarevic & Erdfelder, 2014; Oppenheimer, 2004).

### Source dissociation

According to the source dissociation account, people may forget the original source of a statement (Arkes, Hackett, & Boehm, 1989). In so doing, they might attribute the statement to a source other than the earlier presentation in the experiment, thereby enhancing the effect by increasing its credibility (Arkes et al., 1991, 1989). With source dissociation, participants experience familiarity without conscious recollection of the previous exposure. Like the *sleeper effect* of persuasion (see Kumkale & Albarracín, 2004 for a review), a source dissociation account predicts that the magnitude of the illusory truth effect should increase as memories of contextual details (i.e., source) are lost with time because the original repetition and the accompanying sense of familiarity will be misattributed to a source other than the earlier presentation; people remember the semantic content but not its source.

## The Illusory Truth Effect over Time

In sum, each of the above explanations of the illusory truth effect predicts that the effect should be sensitive to the length of the delay between repetitions. Whereas the source dissociation account hypothesizes a larger illusory truth effect over time as contextual details are lost, the fluency, familiarity, and recognition accounts all predict a decrease in the effect over time because they are enhanced by recency. Note, though, that the fluency account predicts a reduced effect if participants realise that the statements were repeated. All four mechanisms are interrelated and might all contribute to the illusory truth effect synergistically (e.g., fluency and familiarity), antagonistically (e.g., fluency and source disassociation), or individually at different time points. Their precise relationship is an open question (Unkelbach, Koch, Silva, & Garcia-Marques, 2019).

Considered individually, these theoretical predictions are inconsistent with the 2010 meta-analysis that found no relationship between the size of the effect and the delay between repetitions (Dechêne et al., 2010; see ***Table 1***); the magnitude of the illusory truth effect was comparable when repetitions occurred moments apart (Begg & Armour, 1991; Schwartz, 1982) or weeks apart (Arkes et al., 1991, 1989; Bacon, 1979; Gigerenzer, 1984; Hasher, Goldstein, & Toppino, 1977). These results suggest that people can be influenced by repetitions that are both fresh in memory or more stale, and that once a falsehood has been digested, it persists (Lewandowsky, Ecker, & Cook, 2017; Swire, Berinsky, Lewandowsky, & Ecker, 2017).

**Table 1:** Moderator Analysis of Delay Between Sessions (reproduced from Dechêne et al. (2010) meta-analysis).


	SESSION 2	*K*	*D*	95% CI		*Q_b_*

				LOWER BOUND	UPPER BOUND	

Within-items						3.44 (2.74)

	Within day	*9*	*.25 (.24)*	0.07 (0.04)	0.43 (0.46)	

	Within week	*11*	*.44 (.45)*	0.31 (0.29)	0.57 (0.61)	

	Longer delay	*10*	*.44 (.45)*	0.32 (0.28)	0.56 (0.61)	

Between-items						<1 (<1)

	Within day	*25*	*.48 (.49)*	0.39 (0.37)	0.57 (0.62)	

	Within week	*14*	*.43 (.44)*	0.32 (0.28)	0.54 (0.59)	

	Longer delay	*12*	*.48 (.49)*	0.36 (0.32)	0.59 (0.65)	


*Note*: Fixed-effects values are presented outside brackets, and random-effects values are within brackets. Within-items = the difference in ratings for repeated statements between exposure (session 1) and test phase (session 2). Between-items = the difference between truth ratings for new versus repeated statements during the test phase. For within-items, within day is descriptively smaller, however delay did not modify either within-items or between-items as shown by the non-significant goodness of fit statistic *Q_b_*.

Few studies have directly manipulated the interval duration, and the comparisons in the meta-analysis were almost entirely across studies with different participants and tasks. The meta-analysis included only three studies, reported across two papers, that manipulated intersession interval. Both used long delays. One study did not find a difference in the illusory truth effect with intersession intervals of one and two weeks (Gigerenzer, 1984), and another found no difference between one week and one month (Brown & Nix, 1996, Experiment 1). The third study found differences in ratings for false statements after one week, but that effect dissipated after one month and three months (Brown & Nix, 1996, Experiment 2).

Papers published since the 2010 meta-analysis that directly manipulated the interval duration report mixed results. In Nadarevic, Plier, Thielmann, and Darancó (2018, Experiment 2) retention interval (immediately versus 2 weeks) moderated the truth effect when statements were in a foreign language, but did not when the stimuli were in participants’ native language. Nadarevic and Erdfelder (2014, Experiment 1) compared retention intervals of ten minutes and one week within-subjects. They reported no effect with a ten-minute interval, but an effect of *dz* = .54 with a one-week delay. However, Silva, Garcia-Marques and Reber (2017, Experiment 1) observed the opposite pattern between-subjects: *dz* = 1.34 with a few minutes delay and *dz* = .76^1^ after a one-week delay. These studies used different materials and tasks, so the effects of short delays on the illusory truth effect might depend heavily on seemingly minor variations in the study design. In the next section we describe the steps we have taken to minimise such variations and focus on the manipulation of interest.

## Our Experiment

Despite nearly 40 years of research on the illusory truth effect, few studies have systematically varied the effect of intersession delay, and among that subset of studies, little consensus has emerged. Most claims about the effects of delay on the truth effect are based on cross-study comparisons, often of studies using different methods and designs. The lack of direct tests of the effect of delay likely results in part from the challenges of multi-session studies (e.g., ensuring that participants return to the lab multiple times).

Given the limited evidence, conflicting theoretical predictions, and practical importance, we designed a high-powered, preregistered, longitudinal investigation of the illusory truth effect. We systematically manipulated intersession interval in order to directly compare the magnitude of the illusory truth effect produced when statements are repeated (a) immediately, (b) after one day, (c) after one week, and (d) after one month. Systematic measurement of the effect of delay is a prerequisite of conducting more complex studies that build on the assumption that the size of the illusory truth effect is unrelated to intersession interval. For example, when would be the best time to use repetition as a corrective strategy to counter misinformation? We manipulated intersession interval within-subjects (two short and two long delays), using a simple, consistent design across sessions. Based on previous research, we expected to observe the illusory truth effect (i.e., repeated statements rated as subjectively truer) across our two short and two longer delays. Thus, we tested for a main effect of the illusory truth effect, and we examined whether the size of the effect differs across delays:

*H1: We will observe the illusory truth effect. More precisely, we will observe a main effect of repetition averaging across all four delay durations*.

*H2: We will observe a repetition-by-interval interaction such that the size of the illusory truth effect will differ across the delay durations*.

We took several steps to maximise the chances of observing the illusory truth effect. First, we used verbatim repetition of plausible but unknown trivia statements (the “classic” effect). Below, we describe the pre-testing measures we took to ensure that the truth of the statements were unknown to the participants. Second, during the exposure phase, we asked participants to assign each statement to a topic category rather than to judge its truth (Nadarevic & Erdfelder, 2014). Drawing attention to actual truth or falsity of statements at exposure could have reduced or eliminated the size of the effect by encouraging participants to be sceptical when giving their ratings (Brashier, Eliseev, & Marsh, 2020; Jalbert, Newman, & Schwarz, 2020; Nadarevic & Erdfelder, 2014). Similarly, we did not inform participants that half of the items were false as that could have increased scepticism. In the real world, statements typically do not come with such warnings, so including them limits generality (Jalbert et al., 2020).

Our analysis procedures include a number of statistical enhancements as well. Previous work has treated items as a fixed factor, with impaired generalisability as a likely consequence (Judd, Westfall, & Kenny, 2012; Yarkoni, 2019). Studies that neglect stimuli as a potential source of variation can have higher false-positive rates, even if items are counterbalanced (Barr, Levy, Scheepers, & Tily, 2013). We analysed our data using linear mixed-effects modelling which permits the simultaneous modelling of subject and stimulus variability (Baayen, Davidson, & Bates, 2008). Second, given that truth ratings use discrete, Likert-style responses, we used ordinal logistic regression (a cumulative link mixed model) rather than an analysis that assumes those responses were based on an underlying continuous measure (e.g., t-tests, ANOVA, linear regression). Treating ordinal data as continuous can increase the rate of false positives or reduce power, especially in factorial designs where interactions are of primary interest (Liddell & Kruschke, 2018).

We report how we determined our sample size, all data exclusions (if any), all manipulations, and all measures (Simmons, Nelson, & Simonsohn, 2012). Our preregistration, stage 1 manuscript, materials, and data are available on the Open Science Framework at *https://osf.io/nvugt/*.

## Method

### Participants

Participants were recruited online via Prolific and tested using Qualtrics. For recruiting purposes the experiment was described as a study about assessing a range of trivia statements. The full study description used on Prolific can be found in the “Materials & Procedures” component on the OSF.

Participants received the following compensation: Phases 1 and 2 – a total of £3.00 paid after phase 2, phase 3 – £1.00, and phase 4 – £1.00. In addition, participants who completed the entire study received a bonus payment of £1.00. We used the payment structure to motivate participants to continue with the study by paying them for phases 1 and 2 after completion of phase 2. As preregistered, we have included all participants in our analyses who passed the exclusion criteria detailed below, regardless of whether they completed all phases of the study. The final sample that completed all phases of the study without exclusion was n = 507, M_age_ = 37.6 (see ***Table 3***).

At the outset, we used Prolific’s prescreening settings to ensure that participants had an approval rating of ≥99%, had completed at least 20 previous Prolific submissions, listed English as their first language and United Kingdom as their nationality, and were aged between 18 and 65 years. Participants who completed the first phase of the study were listed on a custom allow-list. This Prolific feature enabled us to invite only those participants who took part in the first phase to complete the remaining phases.

### Design

The design consists of two within-subjects factors: 2 (repetition: new vs. repeated) × 4 (retention interval: immediately vs. 1 day vs. 1 week vs. 1 month).

### Sampling Plan

#### Smallest effect size of interest (SESOI)

We planned to balance false-negative and false-positive rates by setting power to 95% and establishing a Type I error rate (alpha) of 5%. Our dependent variable was a Likert-type scale ranging from 1 (*definitely false*) to 7 (*definitely true*). Given the limited previous evidence about the effect size of the interaction between time and repetition, we powered to detect a small time-by-repetition interaction: namely, a difference in illusory truth effect no smaller than a tenth of a scale point across two arbitrarily chosen intervals. This difference equates to about 0.14 on the log odds scale. We chose this threshold as it represents a conservative scenario for detecting an interaction. For instance, if the effect only emerged at the last time point, we could detect that effect with high probability as long as the difference in truth judgements between repeated and new statements was at least a tenth of a scale point larger at that time point than at any other time point. This conservative approach also allowed us to detect a wide variety of other patterns, such as a gradually increasing or decreasing repetition effect, or an effect that reaches asymptote at the second time point, so long as the variance these patterns introduce was at least as large as our SESOI.

#### Comparing our SESOI to previous research

A tenth of a scale point equates to a Cohen’s *d* of approximately 0.20, or a *dz* of approximately 0.34 (with the caveat that this estimate was made possible by treating the ratings data as continuous rather than discrete, and treating stimuli as fixed rather than random). For context, we considered the 17 studies published since the 2010 meta-analysis that used a 6 or 7 point Likert-type scale. The range of scaled raw effects was [0.13, 1.30], with a mean of 0.45. Thus our SESOI of a tenth of a scale point (about 0.14 log odds) was on the bottom of the distribution of reported effects, smaller than the smallest raw effect reported in this literature.

#### Power analyses

Our power analyses, as well as our main analyses, were performed using R 3.6.2 (R Core Team, 2019). We used Monte Carlo simulation to estimate power, simulating data based on estimates for the variance components from an ordinal logit re-analysis of Nadarevic and Erdfelder (2014, Experiment 1; retrieved from the OSF *https://osf.io/eut35/*). Our re-analysis differed from Nadarevic and Erdfelder’s analysis in that we fit a cumulative link (ordinal) mixed model using the ordinal package in R, version 2019.4-25 (Christensen, 2019), which included participants and stimuli (statements) as random factors. The random effects included by-subject and by-stimulus random intercepts and by-subject and by-stimulus random slopes for interval (immediately, 1 day, 1 week, 1 month) and repetition (repeated, new) and their interaction. A report of our re-analysis that includes R code is available in the “Reanalysis of Nadarevic & Erdfelder (2014)” component in the OSF project.

Our analysis confirmed all of their findings except that we did not detect a main effect of the statements’ actual truth or falsity on truth ratings, which suggests that the effect described in the original study may be an artefact of unmodelled stimulus variance. Both in Nadarevic and Erdfelder’s study, and in the present study, the stimuli are pre-tested to be of uncertain truth or falsity to participants. Thus, an effect of actual truth was neither expected, nor the experimental focus. We therefore excluded statement truth or falsity in our analyses. We retained the parameter estimates and wrote code to simulate new data on a seven-point scale based on these values (see the OSF repository for details).

**Figure 1:**
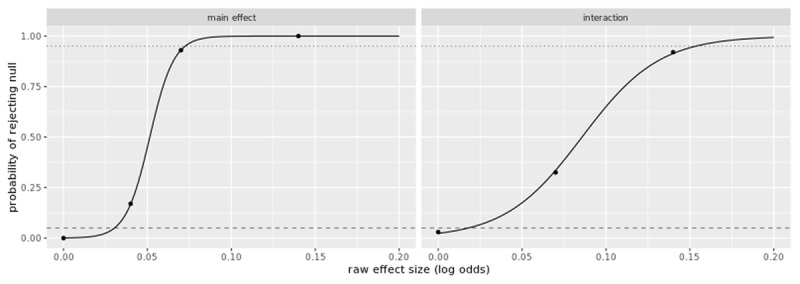
Estimated sensitivity curves for a sample with a final N of 440 participants (based on a starting N of 608 with dropouts). Each point in the plot was based on 100 simulations, and the curves were obtained by fitting a logistic regression model to the data. For reference, the dashed line is at the 5% null rejection rate and the dotted line is at the 95% rejection rate.

Although we were able to estimate a model with maximal random effects (Barr et al., 2013) on the Nadarevic and Erdfelder (2014) data, doing so for our more complex 2 (repetition: new vs. repeated) × 4 (retention interval: immediately vs. 1 day vs. 1 week vs. 1 month) design, in which all factors are both within subject and within stimulus, would have been computationally infeasible given the large number of parameters to estimate. A 2 × 4 design implies eight fixed effect estimates: intercept, effect of repetition, three predictors to code the effect of interval, and three more to code the repetition-by-interval interaction. A maximal random effects model would imply estimation of 64 random effects parameters corresponding to those eight fixed effects, 16 variances (8 for subjects and 8 for stimuli) and 48 covariances. We simplified the model based on the finding that when performing a significance test for some effect of interest, a model that includes only those random slopes related to that effect performs as well as a maximal model (Barr, 2013). We were able to confirm through simulation that using such a ‘minimally sufficient’ specification within the cumulative logistic mixed-effects modelling framework maintained the false positive rate at a nominal level (see ***Figure 1*** below for power simulation results with an effect size of zero). Following this approach, for the test of the interval-by-repetition interaction, the only random slopes included in the model were the by-subject and by-statement random slopes for the three predictors coding the interaction. For the test of the main effect of repetition, the only random slopes included in the model were by-subject and by-statement random slopes for the single predictor coding that factor.

Preliminary benchmarking of our simulation code determined that estimating cumulative link mixed models was too slow to be used to search for plausible subject Ns for power; estimation of a single model on a large simulated data set took as long as 36 hours when the model included only those random effects required to maintain nominal error rates for the interval-by-repetition interaction. Given this limitation, we adopted the strategy of seeking initial estimates of the sample N needed to power the interaction to 95% using a linear-mixed effects model instead of the ordinal model. We then confirmed these initial estimates using the ordinal model. The sensitivity curves in ***Figure 1*** show that we have 95% power to detect an effect of at least .07 (on a log odds scale) for the main effect (H1) and at least 0.14 for the interaction (H2). Further details about the power simulations can be found in the OSF repository under the “Power Calculation” component.

#### Sampling plan

We used Prolific to recruit participants to the study. Following discussions with Prolific, our simulated data assumed a participant attrition rate of 5% between phases 1 and 2, 10% (of the remaining N) between phases 2 and 3, and a further 10% (of the remaining N) between phases 3 and 4. Based on the parameters from the Nadarevic and Erdfelder (2014) data, the attrition rates described above, predicted data loss due to exclusions, and 128 stimuli, the number of participants required to detect either effect with the linear mixed-effects model was 608 participants^2^ at phase 1, and at least 440 participants who provided useable data for the final phase after exclusions. Note that for counterbalancing purposes, the number of subjects starting the study must be a factor of eight. See ***Table 3*** for details of the n recruited, excluded and analysed at each phase.^3^

### Materials

The trivia statements used in our experiment were drawn from those used in two previous illusory truth effects papers, De keersmaecker et al. (2019) and Nadarevic and Erdfelder (2014). The 120 statements used by De keersmaecker et al. (Experiment 3) were originally compiled by Unkelbach and Rom (2017) and were provided via email by Jonas De keersmaecker (05 August, 2019). We amended spellings from US to UK English, corrected misspellings, and excluded statements that were unclear or were not clearly true or false, leaving 95 statements. The 176 statements used by Nadarevic and Erdfelder were retrieved from the OSF (*https://osf.io/eut35/*). We translated those items from German to English using Google Translate, and then asked a native German speaker to check the translations. We excluded statements that we could not translate, that were not clearly true or false, or that were specific to German participants, leaving 145 statements. The combined set of 240 items included 124 false and 116 true statements, and covered a wide range of domains (e.g., history, geography, science).

#### Pre-testing materials

To ensure that the truth of the statements would generally be unknown to UK participants, we pre-tested them using 78 UK participants recruited via Prolific. Statement pre-testing was conducted prior to submitting the stage 1 Registered Report. All materials, data and code from pre-testing can be found in the “Materials & Procedures” component of the OSF project. All analyses were written using R (R Core Team, 2019) and the tidyverse suite of packages (Wickham et al., 2019). We used Prolific’s prescreener settings to select participants with an approval rating of ≥95%, aged between 18 and 65 years, who listed English as their first language and United Kingdom as their nationality.

We randomly split the 240 statements into four sets of 60 (each including 29 true and 31 false statements). Participants were randomly assigned to one of the four sets and statements were presented in random order. Each set of statements was evaluated by 19–21 participants using the same scale as the experimental dependent variable – a Likert-type scale ranging from 1 (*definitely false*) to 7 (*definitely true*). The experiment was self-paced and took approximately 8–10 minutes to complete. Upon completion of the experiment, participants received a payment of £1.30. We excluded data from one participant who self-reported using technical aids to find answers to the question (final sample: N = 77, M_age_ = 33.0). We then selected the 64 true statements and the 64 false statements with mean truth ratings closest to the centre of the scale (i.e., the ones that participants were least certain were true/false).

The resulting final set of 128 trivia statements contained 57 statements from De keersmaecker et al. (2019) and 71 from Nadarevic and Erdfelder (2014), with truth ratings ranging from *M* = 3.50 to *M* = 4.53. The statements were randomised into eight stimulus sets of 16 items that were counterbalanced across participants and across judgement phases (see the Materials & Procedures component for the stimulus lists, code, and csv file). Each set included eight true (e.g., “The area between the eyebrows is called the Glabella”) and eight false (e.g., “A galactic year takes 2500 terrestrial years”) statements. During all phases of the experiment, statements appeared on the screen one at a time, with the rating scale positioned directly below the statement.

### Procedure

The experiment comprised four phases. Phase 1 followed a typical illusory truth effect procedure: Participants read statements in an initial exposure phase, and then rated the truth of a set of statements during a test phase. A screen recording of phase 1 is available in the “Materials & Procedures” component on the OSF (Heycke & Spitzer, 2019).

#### Phase 1

During the exposure phase participants read the 64 statements (half true, half false) that were repeated over the course of the longitudinal experiment. The statements were presented in a different randomised order for each participant. Previous research suggests that if participants rate truth during the exposure phase, they may try to give consistent ratings during the test phase (Nadarevic & Erdfelder, 2014). Rather than asking participants to rate the truth of each item, we ensured that they read the items by asking them to assign each statement to a topic category. Response options were: (1) Art & Entertainment, (2) Geography, (3) History & Politics, (4) Language, (5) Science, Nature & Technology, and (6) Sports.

Immediately afterward, participants completed the first test phase. Participants saw 16 new statements and 16 old statements repeated from the initial exposure phase. For both the new and old statements, half were true and half were false. These 32 statements were presented in a different randomised order for each participant. Participants were asked to judge the truth of each statement on a Likert-type scale ranging from 1 (*definitely false*) to 7 (*definitely true*). Finally, participants completed demographic information about their age, gender, first language, and nationality. They also reported any technical difficulties and whether they had looked up answers. Phase 1 of the experiment took about 15 minutes to complete and, like all phases, was participant-paced throughout.

#### Phases 2 to 4

In the three further phases, participants completed only the test phase from the initial session: One day later, in phase 2, participants read 16 new statements and 16 repeated statements (from the initial exposure phase), and rated each on the same 7-point scale. This procedure was followed in phase 3 after one week, and phase 4 after one month. In every phase the statements were presented in a different randomised order for each participant. The 32 items used in each test phase were sampled without replacement, so each repeated statement appeared in only one of the four test phases, and the new items were not repeated. Participants reported demographic information only after the first session, but reported any technical difficulties and whether they had looked up answers after each phase. Phases 2–4 each took approximately 6–8 minutes to complete.

To increase the chances of participants completing all four phases of the experiment, we allowed some flexibility in the retention intervals: For the one-day condition, participants were able to complete the session between 08.00 and 22.00 the following day. For the one-week condition, participants were able to complete the session seven to eight days after phase 1. For the one-month condition, participants were able to complete the session 28 to 32 days after phase 1.

Debriefing took place at the end of phase 4. The timing of the debrief was explained at the end of each test phase, and contact details for the first author were provided. For participants who dropped out over the course of the experiment, we used their Prolific IDs to send them the debrief after all data collection had been completed. If participants self-reported looking up the answers to questions during any phase of the study, they automatically were directed to a modified debrief that explained that they would not be invited to take part in further phases of the study.

Prior to the full phase 4 debrief, we used funnel debriefing to check whether participants had guessed the broad purpose of the study. These questions were not preregistered but came after all phases of the study were complete, so did not interfere with the experimental design. We asked participants the following sequence of questions: “Do you have an idea about what we were testing in this study? (If yes, please describe what you think we were testing.)”; “Did you notice that some statements were repeated during the study?”; and “Why do you think we repeated some statements?”. Participants then saw the full debrief.

Following the phase 4 debriefing we asked participants to share their views on the future of this research area using two optional questions: Participants were asked an open-ended question about the topics researchers should focus on and they were asked to rank the importance of five prespecified topics (see “Materials & Procedures/FutureResearch”). These questions were purely exploratory; the results are shared in the supplement at *https://osf.io/9zwca/*.

## Analysis Plan

### Outcome-Neutral Criteria

Below we discuss the outcome-neutral criteria that relate to the fidelity of our manipulation and the associated hypothesis tests.

#### Manipulation checks

Since the only prerequisites to the classic illusory truth effect are that plausible but unknown trivia statements are repeated verbatim, a manipulation check was unnecessary for the present experiment. While recognition and processing fluency are the primary explanations for the illusory truth effect, neither are theoretically specified preconditions. Furthermore, the process of attributing fluent processing of a stimulus to truth is considered to be automatic (Bornstein & D’Agostino, 1992), and the experience of fluency is “at the periphery of conscious awareness, resulting in a vague or “fringe” experience of ease” (Reber, Fazendeiro, & Winkielman, 2002, p. 3). Accordingly, fluency is typically studied via its influence on judgements, such as truth, rather than asking participants directly (Reber et al., 2002). In respect to recognition, the effect occurs even when people cannot recall encountering the statements previously (Begg et al., 1992), and conscious recognition might reduce the effect (Alter & Oppenheimer, 2009; Oppenheimer, 2004). Thus self-report measures of perceived processing fluency or recognition are not informative in this case. Also, in a longitudinal design, such checks might have revealed the purpose of the study or have caused participants to respond differently in future phases.

Instead, central to the fidelity of this study was that participants should not know whether each statement was true or false. As reported in the Materials section, we pre-tested the statements and selected those that participants rated closest to the centre of the scale (range *M* = 3.50 to *M* = 4.53). Thus, the statements should have been sensitive to the repetition manipulation, and there was no indication that floor or ceiling effects would occur. Additionally, we excluded any participants who responded uniformly to all statements (see Exclusion Criteria below). This exclusion criteria served as a general attention check that reduced the possibility that participants had not seen the statements before when they were repeated in later phases.

#### Missing data

Given the longitudinal nature of our experiment, we took several steps to help reduce missing data and avoid loss of power. First, as detailed in the Sampling Plan, we planned to collect data until we had usable data from our target N of 440 participants at phase 4. We exceeded our target of 440 because attrition was lower than expected. Second, within the Qualtrics settings we selected “forced response” for all statements to ensure that participants gave ratings for every statement. Third, when recruiting participants we: (a) wrote a clear study description for Prolific explaining that the study involved four phases; (b) used Prolific’s prescreening settings to select participants who were likely to remain active on the site (i.e., those with more than 20 submissions completed and an approval rate of ≥99%; (c) paid a fair hourly rate plus bonus for completing all four phases; and (d) minimised the attrition rate between phases 1 and 2 by paying participants upon completion of phase 2.

We included data from participants who did not complete all four phases. As a form of mixed-effects regression, cumulative link mixed models gracefully handle missing data and thus avoid any need for listwise deletion or data imputation.

### Analytic Reproducibility

The analysis script was written in R Markdown (Allaire et al., 2020). All analyses used R version 3.6.2 (R Core Team, 2019) with the following add-on packages: tidyverse 1.3.0 (Wickham et al., 2019) for data wrangling and visualisation, ordinal 2019.12.10 (Christensen, 2019) for fitting cumulative link mixed models, emmeans 1.4.5 (Lenth, 2020) for follow-up analyses and equivalence tests, and rmarkdown 2.1 (Allaire et al., 2020) for compiling the analysis script. To ensure reproducibility, we created an R package truthiness 1.2.4 (available in the repository or via *https://cran.r-project.org/web/packages/truthiness/index.html*) with functions preprocess() to pre-process and anonymise the raw data, and reproduce_analysis() to re-compile the master R Markdown analysis script (we also included an R Markdown template in the package). Finally, we prepared a Singularity 3.5 software container (*https://sylabs.io*) to ensure that all analyses were performed with the appropriate software versions.

Raw data and code are available in the project repository. We matched data from the same participant across all phases of the study using their Prolific ID. To preserve anonymity, Prolific IDs were removed before we shared the raw data.

## Results

### Exclusion Criteria

Our exclusion criteria were split into participant-level and phase-level exclusions. ***Table 2*** and the list below illustrate the sequence in which exclusion criteria were applied in our data preprocessing code (see the illusory-truth-analysis R Markdown template available through the truthiness package or in the “Analytic Code” component of the OSF). Therefore, if a participant could be excluded for multiple reasons, the reason for exclusion was coded as the first applicable exclusion criterion.

**Table 2:** Sequence of Application of Preregistered and Non-Preregistered Exclusion Criteria.


APPLICATION ORDER	EXCLUSION CRITERIA

Participant-level	

1	Duplicate sessions recorded*

2	Consent to data collection across all four phases was absent*

3	English not first language

4	Used technical aids to answer question(s)

5	Responded uniformly across an entire phase of the study

6	Failed to complete all phases in a reasonable amount of time

7	No ratings data*

8	Other: participant asked for their data to be withdrawn*

Phase-level	

9	Consent for phase was absent*

10	Failed to complete all of the ratings in the phase


*Note*: Non-preregistered criteria are marked with an asterisk. “No ratings data” means that there was no more data left for that subject following application of the phase-level exclusion criteria. This occurred if, for example, a participant partially completed phase 1 before dropping out. Data for that phase would be excluded based on the phase-level criterion “Failed to complete all of the ratings in the phase”, leaving no ratings data for that participant at all, and so we also deleted their participant-level information.

**Table 3:** Participants Recruited, Excluded, Retained, and Analysed, Separated by Experimental Phase and Gender.


PHASE	GENDER	N RECRUITED	N EXCLUDED	N RETAINED	N ANALYSED

1	Female	386	6	380	364

	Male	212	8	204	198

	Gender variant	2	0	2	2

	Prefer not to say	3	0	3	3

	(Missing)	28	28	0	0

	**TOTAL**	631	42	589	567

2	Female	365	10	355	346

	Male	201	2	199	194

	Gender variant	1	0	1	1

	Prefer not to say	3	0	3	3

	(Missing)	4	0	4	0

	**TOTAL**	574	12	562	544

3	Female	347	7	340	337

	Male	197	1	196	191

	Gender variant	1	0	1	1

	Prefer not to say	3	0	3	3

	(Missing)	3	0	3	0

	**TOTAL**	551	8	543	532

4	Female	329	7	322	322

	Male	192	9	183	183

	Gender variant	0	0	0	0

	Prefer not to say	2	0	2	2

	(Missing)	3	0	3	0

	**TOTAL**	526	16	510	507


*Note*: “Missing” refers to participants who did not finish phase 1 and therefore did not report their gender. Four of these participants were erroneously invited back to future phases because they started multiple sessions at phase 1. Participants who started multiple sessions during any phase were excluded from analyses. “N retained” is the number of participants after exclusions were applied at the end of each phase. “N analysed” is the number of participants after exclusions were retroactively applied. For example, if a participant responded uniformly to all statements during phase 4, their data were excluded from all previous phases.

We excluded all data from any participant who did not meet the following criteria during *any phase* of the study: self-reported having any language other than English as their first language (n = 11), self-reported using technological aids to answer question(s) (n = 3), or who responded uniformly (e.g., always answer 1) to all topic categorisations (phase 1 only) or to all truth ratings (in any phase; n = 7). To account for overly fast or slow completion of the study in the absence of an experimenter observing data collection in person, we excluded participants who completed the study in more than 40 minutes (for phase 1, and 30 minutes all other phases), or less than 3 minutes (for phase 1) and 1 minute (all other phases; n = 22). In addition to the preregistered exclusion criteria, we excluded participants who, during phase 1, did not consent to complete all four phases (n = 10), or who started duplicate sessions (n = 8), and we manually excluded a participant who requested that their data be withdrawn (n = 1). We also removed participant-level information for participants who had no ratings data left after applying all phase-level exclusions (n = 2). In total, the participant-level exclusion criteria resulted in the removal of 64 participants from the sample.

Phase-level exclusions were applied after any participant-level exclusions (i.e., on phases that remained after removing data from those 64 participants). At the phase level, we excluded data from any participant who elected to end their participation prior to completing an entire phase of the study (n = 13; see ***Table 3***). For example, if a participant elected to end phase 3 of the study before completing it, we retained their data for phases 1 and 2 of the study but not for phases 3 or 4. In addition to the preregistered exclusion criteria, we excluded data from phases where a participant did not provide consent (n = 1). In total, the phase-level exclusion criteria resulted in the removal of 14 phases.

Retention was better than anticipated across all phases of the study. The combination of exclusions and dropouts resulted in 567 total participants providing data for at least one phase; of the 526 participants who attempted phase 4, data from 507 were analysed (***Table 4***).

**Table 4:** Summary of Exclusions, Dropouts, and Attrition by Phase.


PHASE	RECRUITED	ATTEMPTED	EXCLUDED	RETAINED	ANALYSED	DROPOUT	EXCLUDED	ATTRITION

1	NA	631	42	589	567	NA%	10.1%	NA%

2	589	574	12	562	544	2.5%	5.2%	7.7%

3	566	551	8	543	532	2.7%	3.4%	6.1%

4	545	526	16	510	507	3.5%	3.6%	7.1%


*Note*: “Retained” is the number of participants after exclusions were applied at the end of each phase. “Analysed” is the number of participants after exclusions were retroactively applied. For example, if a participant responded uniformly to all statements during phase 4, their data were excluded from all previous phases.

### Confirmatory analyses

After importing the data and applying the exclusion criteria, we fitted a cumulative link mixed model to the data using clmm() from the ordinal package (Christensen, 2019) which allowed us to model the effects of repetition, interval, and the repetition-by-interval interaction in log-odds space, with a set of thresholds (cut points) representing the ordinal response categories. We fit models using ‘flexible’ thresholds (the default), which allows the distance between the six cut-points making up the seven point scale to vary freely. This proved the best fitting approach on the Nadarevic and Erdfelder (2014) data. The “Simulated Data & Analyses” component of the project repository contains HTML reports with results from applying the analysis script to data simulated under three different hypothetical scenarios: null main effect and null interaction (analysis_all_null.html), significant main effect and null interaction (analysis_main_effect.html), and significant main effect and significant interaction (analysis_interaction.html). Please consult the repository for further details, including the full annotated code and an appendix with information on how to reproduce the results.

The fixed effects of repetition (new, repeated) and interval (immediately, one day, one week, one month) were coded using deviation-coded numerical predictors. As described above, the models included participants and stimuli as random factors, and the minimally sufficient random slopes for the fixed effect being tested. Each of the two hypotheses were tested using likelihood ratio tests, comparing models with and without the fixed effect or effects of interest, with all random effects held constant. As laid out in the supplementary materials, we started with descriptive statistics and a visualisation of the results, followed by inferential statistics. We followed the preregistered plan summarised in ***Table 7***; using test 1 to test the main effect (H1), and tests 3 and 4 to test the repetition-by-interval interaction (H2).^4^

#### Test of main effect (H1)

We tested the main effect of repetition using a *χ*^2^ test with one degree of freedom, and with α = .05 (***Table 7***, test 1). Supporting H1, there was a significant main effect of repetition when collapsing over interval, \widehat {{\beta _R}}\, = \,0.57 (*SE* = 0.04), *χ*^2^(1) = 171.88, *p* < .001. Ratings averaged across items and participants were higher for repeated statements (*M* = 4.51, *SD* = 1.45) than those for new statements (*M* = 4.13, *SD* = 1.34).^5^

#### Test of repetition-by-interval interaction (H2)

**Figure 2:**
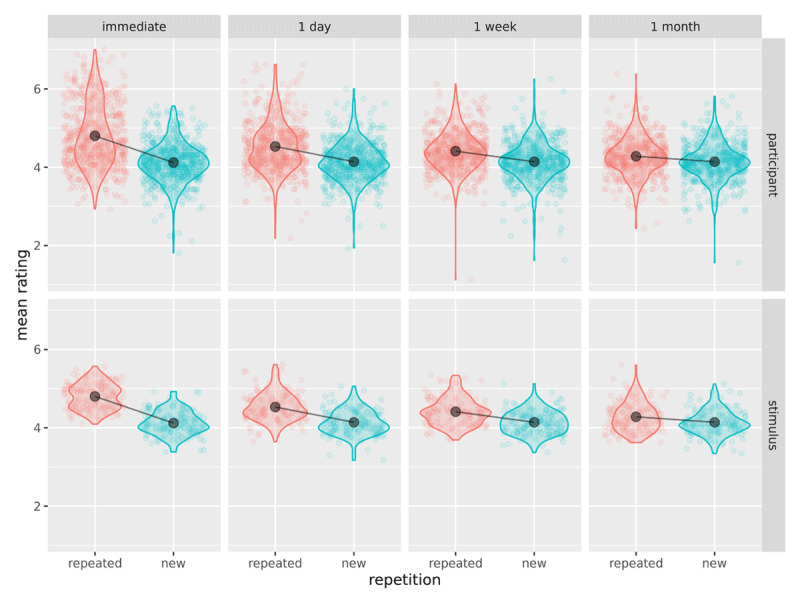
Effect of repetition across interval, cell means (black points, line) plotted against participant means (top row) and stimulus means (bottom row).

Next, we tested the repetition-by-interval interaction using a *χ*^2^ test with three degrees of freedom and α = .05 (***Table 7***, test 3). There was a significant repetition-by-interval interaction, {\hat \beta _{R:I1}}\,\, = \,\,-0.47 (*SE* = 0.05; immediately vs. one day), {\hat \beta _{R:I2}}\,\, = \,\,-0.67 (*SE* = 0.07; immediately vs. one week), {\hat \beta _{R:I3}}\,\, = \,\,-0.84 (*SE* = 0.07; immediately vs. one month), *χ*^2^(3) = 121.15, *p* < .001. This significant interaction supports H2, indicating that the illusory truth effect varies over time. The size of the illusory truth effect decreased over time; the difference between the two ratings (repeated minus new) decreased as the interval increased (see ***Table 5***). The data showed greater variability across participants than across stimuli (***Figure 2***).

**Table 5:** Mean Ratings and SDs for Repeated Versus New Statements, and Their Difference, by Interval.


INTERVAL	REPEATED M (SD)	NEW M (SD)	DIFFERENCE

immediately	4.80 (1.54)	4.12 (1.36)	0.68

1 day	4.53 (1.45)	4.14 (1.36)	0.39

1 week	4.41 (1.38)	4.14 (1.33)	0.27

1 month	4.28 (1.37)	4.14 (1.32)	0.14


We followed this significant result using the function emmeans() to attempt to localise the effect, testing the effect at each of the four intervals, and using a Holm-Bonferroni stepwise procedure (Holm, 1979) to keep the familywise error rate at .05 (***Table 7***, test 4). Pairwise comparisons revealed that at every interval, estimated marginal means for repeated statements were significantly higher than those for new statements, indicating that the illusory truth effect was present at all four phases (***Table 6***).

**Table 6:** Planned Comparisons of the Simple Effect of Repetition at Each Interval, with Holm-Bonferroni Correction.


CONTRAST	INTERVAL	ESTIMATE	SE	Z RATIO	REJECT NULL

repeated – new	Immediately	1.04	0.05	22.68	True

repeated – new	1 day	0.56	0.03	18.64	True

repeated – new	1 week	0.37	0.03	11.00	True

repeated – new	1 month	0.20	0.04	5.53	True


**Table 7:** Summary of Experimental Design from Research Questions to Results.


QUESTION	HYPOTHESIS	TEST NO	ANALYSIS PLAN	POWER ANALYSIS	RESULTS

Is there a time-invariant illusory truth effect?	H1: We will observe a main effect of repetition averaging across all four delay durations.	1	Fit a cumulative link mixed model (as detailed in the “Simulated Data & Analyses” component on the OSF) and conduct *χ*^2^ test with one degree of freedom, with α = .05.	95% power to detect an effect of .07 or larger on the log odds scale (about a twentieth of a scale point on a seven-point scale). Based on 440 participants completing phase 4.	Supporting H1, there was a significant main effect of repetition when collapsing over interval, \widehat {{\beta _R}}\,\, = \,\,0.57 (*SE* = 0.04), *χ*^2^(1) = 171.88, *p* < .001.

		2	IF tests 1 and 3 are non-significant: Test for the absence of the main effect using an equivalence test with bounds of Δ_L_ = –0.14 and Δ_U_ of 0.14 on a log odds scale.	95% power to reject the null of a raw effect greater than .085.	

Does the illusory truth effect vary over time?	H2: We will observe a repetition-by-interval interaction such that the size of the illusory truth effect will differ across the delay durations.	3	Fit a cumulative link mixed model (as detailed in the “Simulated Data & Analyses” component on the OSF) and test the repetition-by-interval interaction using a *χ*^2^ test with three degrees of freedom and α = .05.	95% power to detect an effect of a tenth of a scale point, (about.14 on the log odds scale) between two arbitrarily chosen time points: If an illusory truth effect only emerges at very the last time point, we can detect it with 95% power as long as it is at least a tenth of a scale point. Based on 440 participants completing phase 4.	Supporting H2, there was a significant repetition-by-interval interaction, {\hat \beta _{R:I1}}\,\, = \,\,-0.47 (*SE* = 0.05; immediately vs. one day), {\hat \beta _{R:I2}}\,\, = \,\,-0.67 (*SE* = 0.07; immediately vs. one week), {\hat \beta _{R:I3}}\,\, = \,\,-0.84 (*SE* = 0.07; immediately vs. one month), *χ*^2^(3) = 121.15, *p* < .001.

		4	IF test 3 is significant: Use emmeans() to attempt to localise the effect, testing the effect at each of the four intervals, and using a Holm-Bonferroni stepwise procedure to keep the familywise error rate at .05.	N/A	Pairwise comparisons revealed that at every interval, estimated marginal means for repeated statements were significantly higher than those for new statements, indicating that the illusory truth effect was present at all four phases (Table 6).

		5	IF test 3 is non-significant: Test for the absence of an interaction effect using an equivalence test considering all six possible pairwise comparisons of the illusory truth effect across intervals to see whether they fall within the bounds of Δ_L_ = –0.14 and Δ_U_ of 0.14 on a log odds scale	With |Δ| =.14, 37% power to reject H_0_ if the true value is 0, about 18% power if true value is .07 or smaller. With |Δ| =.20, 93% power if the true value is 0, 75% power if the true value is .07 or smaller, 18% power if the true value is .14 or smaller. For results with .14 < |Δ| < .20, see equivtest.html in the repository.	


#### Model validation

**Figure 3:**
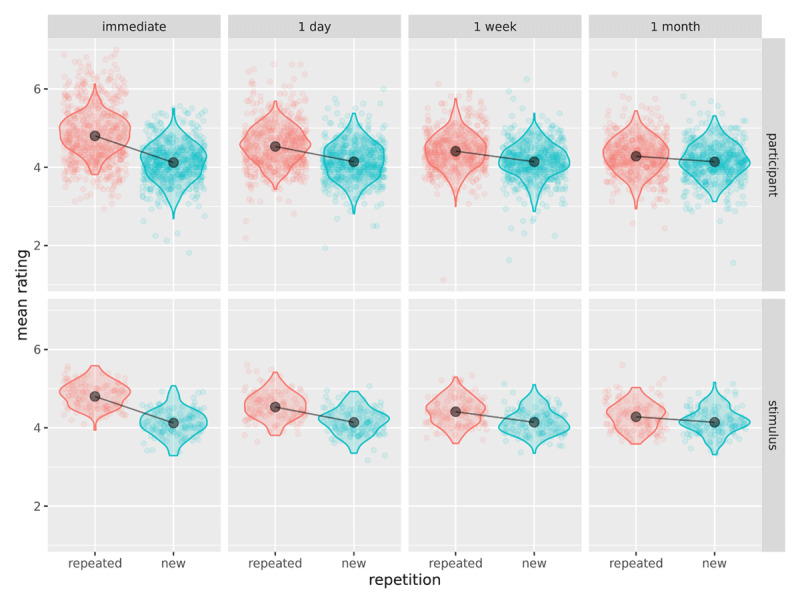
Model validation: Plot of observed participant/stimulus means (points) against simulated data distributions (violins) and cell means (black points, line).

As noted above, our cumulative link mixed-modelling approach makes fewer and more reasonable assumptions about the data as compared to a traditional approach using ANOVA. Unlike ANOVA, the cumulative link mixed-modelling approach does not assume that the data come from a continuous, unbounded scale; nor does it assume equal psychological distances between response categories. This leaves fewer assumptions to be tested than in a conventional ANOVA-based analysis. The main two assumptions behind cumulative link mixed-models are the proportional odds assumption and multivariate normality of the random effects in logit space. Although there has been some discussion of testing the proportional odds assumption for models without random effects (Harrell, 2015) we know of no accepted way to test this assumption for models with random effects. Also, there is no clear consensus in the mixed-modelling literature on how to check the assumption for multivariate normality, with a primary difficulty being distinguishing effects of an ill-fitting model from effects of the data structure or model fitting procedure (Loy, Hofmann, & Cook, 2017). Therefore, we opted to check our model fit using graphical methods. In particular, we generated a reference distribution by simulating data from the model parameter estimates and plotted these distributions against the observed data distributions for participants and stimuli (see ***Figure 3***). Should more systematic model checking procedures be developed in the future, our data are freely available for re-evaluation.

***Figure 3*** suggests a good fit to the data, except that the model underestimates the by-subject variability for repeated statements at the two earliest phases (immediately, 1 day). However, the effects are so large at these two phases that it seems extremely unlikely that a more complex model that accounted for this overdispersion would yield different results.

### Exploratory analyses

We found that fitting ordinal models on such a large dataset required unreasonable amounts of computation time (up to 24 hours per model on a standard desktop computer). For expedience, our exploratory analyses used standard regression models and correlation instead of ordinal models. The analyses primarily focus on factors that might modulate the illusory truth effect: attention during exposure phase, test phase completion time, and participant age.^6^ However, first we explored what proportion of participants showed the illusory truth effect.

#### How many participants displayed the predicted effect?

**Figure 4:**
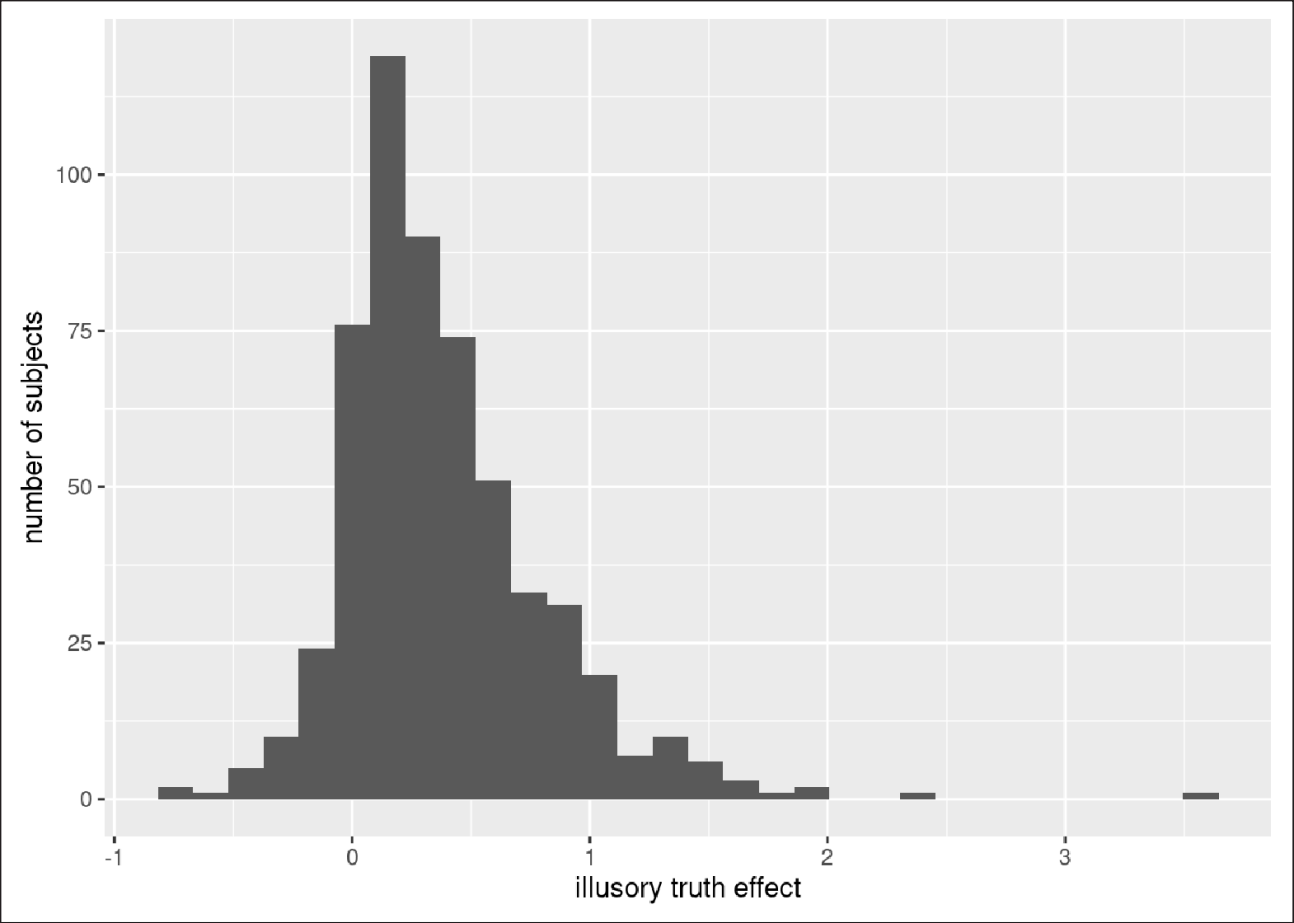
Distribution of participants showing an overall effect of the illusory truth effect.

We were interested in how many participants behaved consistently with the prediction that repeated statements would be rated as truer than new statements (Grice et al., 2020). In order to calculate an overall illusory truth effect for each participant, we combined across all 128 statements and subtracted scores for new items from those for repeated items. Four hundred and eighty-three participants out of 567 (85.2%) showed higher mean truth ratings for repeated statements (see ***Figure 4***). Calculated phase-by-phase, the proportion of participants showing this pattern declined with time: immediately (75%), 1 day (72%), 1 week (71%), and 1 month (61%).

#### Seriousness check

Next, we explored how seriously participants took their participation in the study, and whether seriousness interacted with the illusory truth effect. We used the exposure task—assigning the statements to topic categories—as a proxy for participant seriousness. Each statement had a correct category^7^ (e.g., “A polo game is divided into periods of 7.5 minutes called ‘chukkas’.” would be categorised as “sports”). If participants concentrated during the exposure phase then generally they should have accurately assigned statements to the correct category. One caveat is that some statements might have addressed unfamiliar topics for some participants, meaning that they would have guessed the answer.

**Figure 5:**
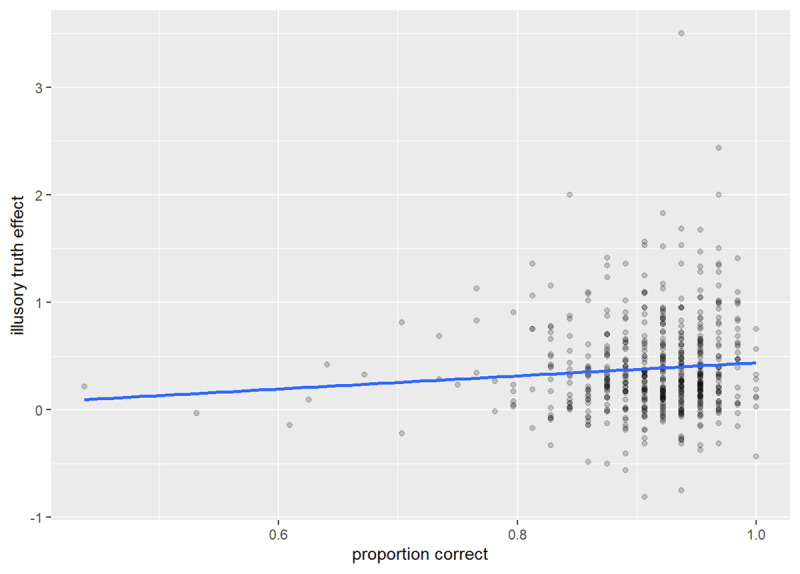
Illusory truth effect by category judgment accuracy.

Generally participants were accurate in their categorisations (*M_proportion correct_* = .912, *SD* = .06; see ***Figure 5***). In a linear model, just 0.53% of the variation in the illusory truth effect was explained by exposure task accuracy, *F*(1, 565) = 3.99, *p* = .046, *R*^2^ = .01. A Spearman’s rho correlation revealed a small correlation between the overall illusory truth effect and exposure task accuracy *r_s_* = .09, *p* = .040. Thus, the seriousness with which participants approached the task was largely unrelated to the size of the illusory truth effect.

#### Phase duration

Next, we investigated whether there was a relationship between the illusory truth effect and how long people took to complete each test phase. Participants who spent longer completing the task might have expended more effort and given higher ratings for repeated statements. Since phase duration varied with interval (i.e., phases 1 and 4 were longer than 2 and 3), we modelled log duration as a function of phase and used the residuals from this model to predict the size of the illusory truth effect. A Spearman’s rho correlation revealed a small correlation between the overall illusory truth effect and phase duration *r_s_* = .04, *p* = .045. However a linear model relating the two parameters was non-significant, *F*(1, 2148) = 1.96, *p* = .162, *R*^2^ < .001. Consistent with the results from the seriousness analysis, there is little evidence that participants’ attentiveness to the task was associated with the size of the illusory truth effect. However, these results should be interpreted with the caveat that our stringent criteria for study participation might have selected for conscientious participants.

#### Age

**Figure 6:**
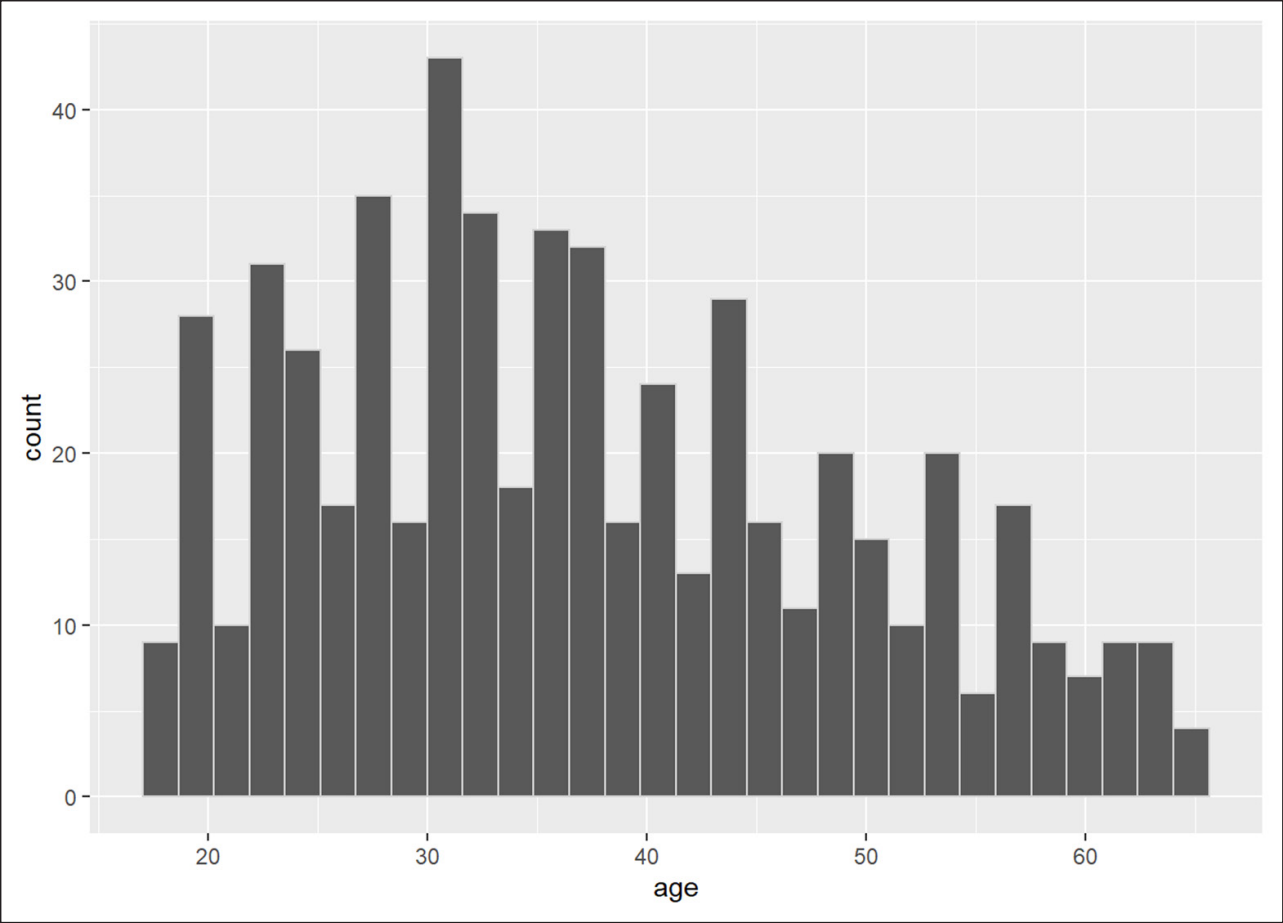
Distribution of participants’ age.

Since all explanations of the illusory truth effect rely on memory, and memory performance varies with age, we investigated whether age modulated either the main effect, or the trajectory of the effect over time. Participant ages ranged from 18 to 65 years, with a mean of 37.24 years (*SD* = 12.08; see ***Figure 6***).

The Spearman’s rho correlation between the overall main effect of illusory truth and age was non-significant, *r_s_* = .06, *p* = 0.154. Similarly, the results of the linear model were non-significant, *F*(1, 565) = 0.54, *p* = .464, *R*^2^ < .001, suggesting little relationship between a participant’s age and the overall size of their illusory truth effect.

**Figure 7:**
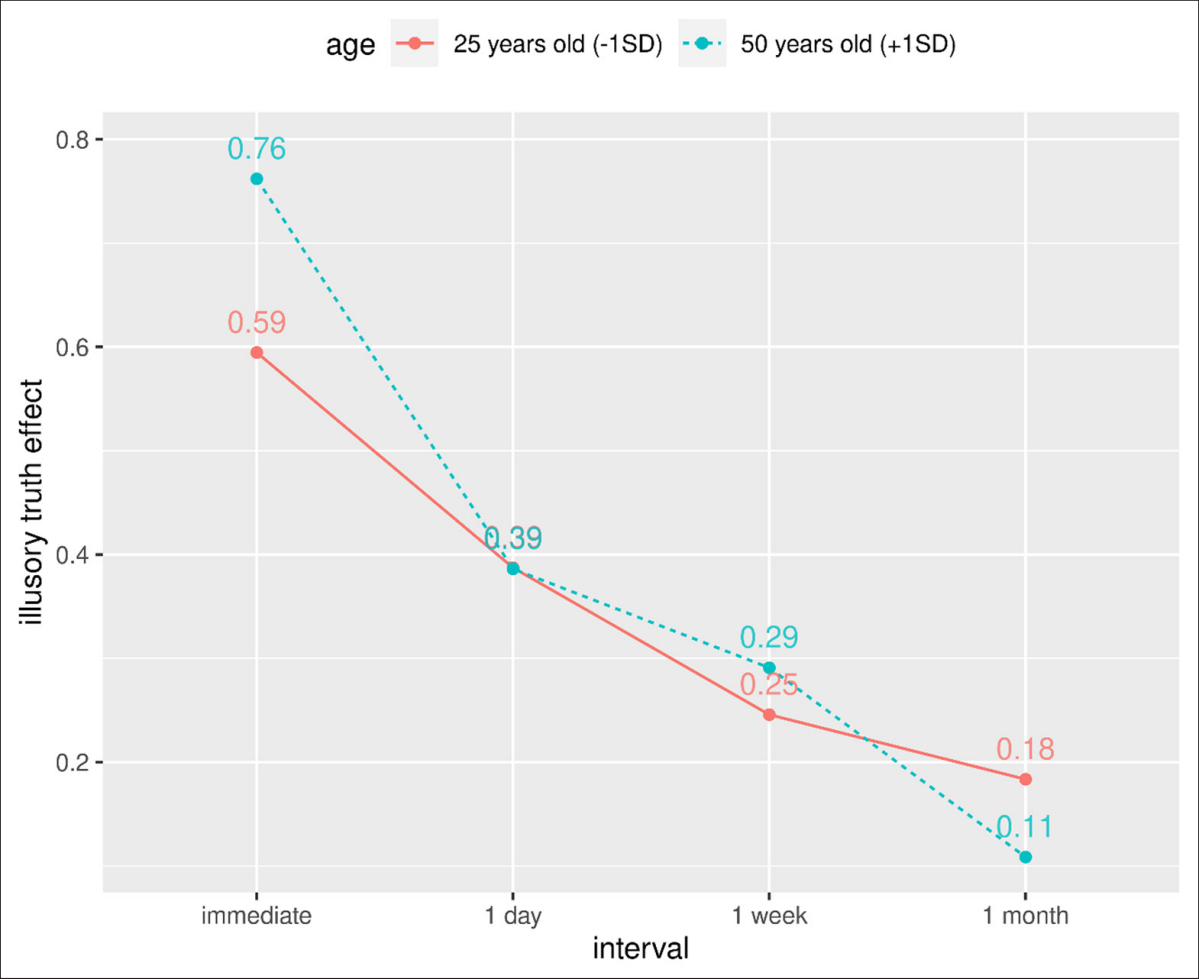
Model predictions for the trajectory of the illusory truth effect for two ages.

Next, we examined whether age modulated the decline in the illusory truth effect over time. The finding of a consistently decreasing trend in the confirmatory analysis above, with no discontinuities or asymptotic behaviour, suggests that the data are amenable to polynomial regression, which provides a more concise mathematical summary of the trajectory than the 2 × 3 factorial analysis we used above. As a first step, we used model comparison to determine whether the trajectory was best described as a linear, quadratic, or cubic polynomial function of interval. A cubic model provided the best fit for our data. We found that variance in trajectory by age was statistically significant, *χ*^2^(3) = 11.08, *p* = .011. To interpret the trajectory-by-age interaction, we modelled the predicted trajectories for two age groups: participants aged 25 years (about 1 SD below the mean age) and participants aged 50 years (about +1 SD above the mean age). As can be seen in ***Figure 7*** the trajectories vary by age, with the primary difference concentrated at the immediate interval, where older participants showed a bigger truth effect (0.76 versus 0.59 for 50 versus 25 year olds, respectively), and at 1 month with older participants showing a smaller truth effect (0.11 versus 0.18, respectively; see ***Figure 7***).

### Supplement

The illusory truth effect has typically been analysed using ANOVAs. We have outlined the reasons that cumulative link mixed modelling is more appropriate for these data. For comparison, we report the results (in the online supplementary materials at *https://osf.io/ngrw7/*) of a 2 (repetition: new vs. repeated) × 4 (retention interval: immediately vs. 1 day vs. 1 week vs. 1 month) repeated measures ANOVA using participants’ mean truth ratings as the dependent variable. Although we did not use this ANOVA for our confirmatory hypothesis test (H2), the results were in line with the findings from our cumulative link mixed model.

Additionally, the online supplement documents further exploratory analyses of statement length and topic, the results of three funnel debrief questions used to ascertain whether participants had guessed the purpose of the overall study, as well as questions about the topics researchers should study in the future.

## Discussion

We used a repeated measures, longitudinal design to investigate the trajectory of the illusory truth effect over time: immediately, one day, one week, and one month. Both of our hypotheses were supported: We observed a main effect of the illusory truth effect when averaging across all four delay conditions (H1). The illusory truth effect was present at all four intervals, but the size of the effect diminished as the interval duration increased (H2). The repeated-minus-new difference was largest when tested immediately (0.67) and shrank after one day (0.39), one week (0.27), and one month (0.14). This reduction in the illusory truth effect over time is inconsistent with an earlier meta-analysis that found no relationship between the size of the effect and intersession interval across studies (Dechêne et al., 2010), but it is consistent with one between-subjects study showing a smaller effect after one week than after a few minutes (Silva et al., 2017, Experiment 1).

The reduced effect after a delay is consistent with the recognition, familiarity, and processing fluency explanations of the illusory truth effect. All three explanations predict larger effects for recently repeated items and smaller effects as feelings of recognition, familiarity or fluency fade with time.

A caveat to the processing fluency account occurs when the source of fluency is obvious (e.g., when participants recognise that statements have been recently repeated). In such cases, participants might not use processing fluency to make their judgments of truth, thereby eliminating the effect (Alter & Oppenheimer, 2009; Nadarevic & Erdfelder, 2014; Oppenheimer, 2004). Our results challenge this fluency discounting explanation because the size of the illusory truth effect was greatest when tested immediately, when participants should be most aware that some statements had been repeated. Similarly, the source disassociation hypothesis predicts that the illusory truth effect should increase with time as people forget that they saw the statements during the experiment, remembering only the semantic content and attributing it to a source outside the experiment. Here we find the opposite.

Our exploratory analyses revealed that most participants (85.2%) showed the illusory truth effect, suggesting that the effect is reliable across participants. However, fewer people might show the effect if participants had been warned that some statements would be false. We chose not to do so because in the real world, false statements are not accompanied by warnings (Jalbert et al., 2020).

The overall illusory truth effect was not associated with individual differences in participants’ diligence in performing the task. However, we cannot be sure that participants were attentive the whole time because our online study only provided an overall measure of phase duration. It is possible that participants who spent longer during a phase actually might have been distracted and less attentive.

Our exploratory analyses revealed little association between age and the overall illusory truth effect, but the trajectory of the effect over time did vary with age. Compared to younger participants (25 years), older participants (50 years) showed a bigger truth effect at the immediate interval and a smaller effect at the 1-month interval. If future research replicates this relationship between age and the repetition-by-interval interaction, then this pattern might reflect a reduction in memory in older adults: During the immediate phase, reduced working memory performance might lead to a feeling of familiarity (without explicit recognition) that could increase truth ratings. At one month, a decline in longer term memory could mean that statements are neither recognised nor familiar, resulting in a smaller truth effect.

### Constraints on Generality (COG) Statement

We used Prolific to recruit UK adults (18–65 years) with English as a first language. Given that the illusory truth effect has been observed in a range of countries using a range of languages, we expect the repetition-by-interval interaction to generalise to other WEIRD adults (Western, Educated, Industrialized, Rich and Democratic; Henrich, Heine, & Norenzayan, 2010), regardless of language spoken. However we lack evidence showing that the results will generalise beyond this population. Our pre-screen questions on Prolific might have selected for more conscientious participants, but there is no evidence that personality differences contribute to the illusory truth effect (De keersmaecker et al., 2019).

Given that we modelled stimuli as random effects and show that the illusory truth effect is not reliant on particular trivia items, we expect our results to generalise to other sets of statements that comply with these two critical features: 1) ambiguous veracity—if participants already know the answer to questions, they may use existing knowledge. Future replications or extensions should follow our statement pre-testing procedure to ensure that statements are generally unknown; 2) topic—statements should not relate to dearly held beliefs. Considering that the vast majority of illusory truth effect research uses trivia statements as stimuli, it is not clear that this result will generalise to topics on which participants hold strong prior beliefs. Our exposure task ensured that participants read and processed the statements, but we did not ask participants to rate them for truth. We do not know whether the effect of delay would be robust if participants rated truth at exposure because that might lead them to give consistent responses (Nadarevic & Erdfelder, 2014).

We do not think the effect is likely to rely on the presentation medium (computer vs. paper and pencil), and we have no reason to expect temporal or historical context to be relevant in observing the repetition-by-interval interaction. That said, data collection occurred during the COVID-19 pandemic (December 2020 to January 2021) when the UK was under lockdown or semi-lockdown. The timing of the study might partially account for the high retention across the experiment. We have no reason to believe that the results depend on other characteristics of the participants, materials, or context.

### Future Research

This Registered Report is the first study to manipulate intersession interval systematically over two short and two longer time periods, so the results should be replicated to ensure their generalisability, particularly because theories on the underlying mechanisms of the effect make contradictory predictions about the effect’s trajectory. Future research should also consider the real-world implications of the observed differences and attempt to calibrate them. For example, what is the implication of a 0.67 increase on a 7-point Likert-type scale in a person’s truth judgement compared to 0.14? Given the reduction to a 0.14 difference after one month, future research may also investigate the temporal boundaries of the effect.

## Conclusion

The aim of this study was to investigate whether the size of the illusory truth effect depends on the intersession interval. We found the size of the effect declined over time. Whereas previous research suggested that the effect size was unrelated to the interval between exposure and test phases (Dechêne et al., 2010), our results suggest that researchers should consider the implications of the choice of an intersession interval when drawing inferences about the illusory truth effect. An effect that diminishes with time is consistent with the recognition, familiarity, and processing fluency explanations of the effect: As feelings of recognition, familiarity or fluency decrease, so does the effect of repetition on judged truth. The repetition-by-interval interaction implies that when false information is repeated over short timescales it may have a greater effect on truth judgements than repetitions that are far apart.

## Data Accessibility Statement

The anonymised raw data and code used to analyse the data, are available in the OSF repository *https://osf.io/nvugt/* as well as in the accompanying truthiness package for R *https://cran.r-project.org/web/packages/truthiness/index.html*. Anonymised raw data, code, and materials for the statement pre-testing, along with materials and the lab log from the main experiment can also be found in the OSF repository.

## Footnotes

The effect sizes relate to the new vs. repeated comparison that was manipulated within-subjects, hence they are reported as dz even though retention interval was manipulated between-subjects.

Prolific recruited 631 participants to fulfil our target of 608 participants. The platform continues to recruit participants until the target number of participants have *completed* the experiment.

We removed the following text from the Stage 1 report that described the process we would use to recruit additional participants if we had fewer than 440 useable participants after phase 4: “If at the end of phase 4, and after applying exclusions to all phases, we have usable data from fewer than 440 participants, we will recruit additional new participants. We will calculate the observed attrition and exclusion rate over the course of the study and use this rate to calculate the additional number of participants we should recruit to achieve a sample of about 440 usable participants. The process of recruiting additional participants will continue either until we achieve 440 usable participants, or we have recruited 700 participants to phase 1 of the study, whichever comes first. This maximum participant N is based on funding constraints. In the case that we have usable data from fewer than 440 participants, we will report the smallest effect size that we had 95% power to detect (a sensitivity analysis).”

See the stage 1 Registered Report for a detailed description of the processes we would have followed had the results of tests 1 and/or 3 been non-significant (*https://osf.io/9mncq* p. 22).

Text from the Stage 1 report that considered the possibility of a non-significant outcome has been removed: “By itself, the result of this test is not diagnostic for either hypothesis, because if the illusory truth effect varies over time (repetition-by-interval interaction), this could yield either a significant or non-significant main effect, depending on how the effect is distributed over time. For example, the effect could be countervailed if it appeared at two time points, and was reversed at two time points. Or, it could be underpowered if the effect was present for one interval but not at the others.”

We conducted the age and attention during exposure phase analyses based on feedback from reviewers of our Stage 1 manuscript.

Some statements fit into two categories. Where this was the case, we considered either categorisation to be correct.
